# Automatic gross tumor segmentation of canine head and neck cancer using deep learning and cross-species transfer learning

**DOI:** 10.3389/fvets.2023.1143986

**Published:** 2023-03-21

**Authors:** Aurora Rosvoll Groendahl, Bao Ngoc Huynh, Oliver Tomic, Åste Søvik, Einar Dale, Eirik Malinen, Hege Kippenes Skogmo, Cecilia Marie Futsaether

**Affiliations:** ^1^Faculty of Science and Technology, Department of Physics, Norwegian University of Life Sciences, Ås, Norway; ^2^Faculty of Science and Technology, Department of Data Science, Norwegian University of Life Sciences, Ås, Norway; ^3^Faculty of Veterinary Medicine, Department of Companion Animal Clinical Sciences, Norwegian University of Life Sciences, Ås, Norway; ^4^Department of Oncology, Oslo University Hospital, Oslo, Norway; ^5^Department of Physics, University of Oslo, Oslo, Norway; ^6^Department of Medical Physics, Oslo University Hospital, Oslo, Norway

**Keywords:** canine head and neck cancer, dogs, radiotherapy, automatic target volume segmentation, gross tumor volume, artificial intelligence, deep learning, cross-species transfer learning

## Abstract

**Background:**

Radiotherapy (RT) is increasingly being used on dogs with spontaneous head and neck cancer (HNC), which account for a large percentage of veterinary patients treated with RT. Accurate definition of the gross tumor volume (GTV) is a vital part of RT planning, ensuring adequate dose coverage of the tumor while limiting the radiation dose to surrounding tissues. Currently the GTV is contoured manually in medical images, which is a time-consuming and challenging task.

**Purpose:**

The purpose of this study was to evaluate the applicability of deep learning-based automatic segmentation of the GTV in canine patients with HNC.

**Materials and methods:**

Contrast-enhanced computed tomography (CT) images and corresponding manual GTV contours of 36 canine HNC patients and 197 human HNC patients were included. A 3D U-Net convolutional neural network (CNN) was trained to automatically segment the GTV in canine patients using two main approaches: (i) training models from scratch based solely on canine CT images, and (ii) using cross-species transfer learning where models were pretrained on CT images of human patients and then fine-tuned on CT images of canine patients. For the canine patients, automatic segmentations were assessed using the Dice similarity coefficient (*Dice*), the positive predictive value, the true positive rate, and surface distance metrics, calculated from a four-fold cross-validation strategy where each fold was used as a validation set and test set once in independent model runs.

**Results:**

CNN models trained from scratch on canine data or by using transfer learning obtained mean test set *Dice* scores of 0.55 and 0.52, respectively, indicating acceptable auto-segmentations, similar to the mean *Dice* performances reported for CT-based automatic segmentation in human HNC studies. Automatic segmentation of nasal cavity tumors appeared particularly promising, resulting in mean test set *Dice* scores of 0.69 for both approaches.

**Conclusion:**

In conclusion, deep learning-based automatic segmentation of the GTV using CNN models based on canine data only or a cross-species transfer learning approach shows promise for future application in RT of canine HNC patients.

## 1. Introduction

Head and neck cancer (HNC) is a heterogeneous group of malignant neoplasms originating from the different anatomical sites of the upper aerodigestive tract (1) and is relatively frequent in both humans and dogs. For humans, HNC is the seventh leading cancer by incidence worldwide (2), of which 90 % are squamous cell carcinomas (SCCs) of the oral cavity, oropharynx, hypopharynx, and larynx (3). The incidence rate of HNC in dogs is similar to that of humans, but canine HNC patients present a greater variety of cancer subtypes and SCCs are less predominant than in humans (4–6). For the same cancer subtypes, however, dogs with spontaneous tumors have been used as a comparative species in cancer research, taking advantage of the relative similarity of tumor biology and anatomic size between human and canine patients (7–9).

In humans, the main curative treatment modalities for HNC are surgery, radiotherapy (RT), chemotherapy, or a combination of these. Treatment decisions are typically based on primary tumor site and stage. However, most human HNC patients receive RT as an integral part of treatment (1). At present, the most frequently used RT technique for HNC in humans is intensity-modulated RT (IMRT) (1). IMRT is a high-precision technique, offering highly conformal radiation doses to the target and improved sparing of surrounding critical normal tissue structures, known as organs at risk (OARs), compared to conventional and three-dimensional (3D) conformal RT (10–12). These advantages are highly relevant for the treatment of HNC due to the complex anatomy of the head and neck region with immediate proximity between irradiated target volumes (TVs) and OARs.

In dogs, surgery is the primary treatment for most HNCs, but RT is indicated as the primary treatment for sinonasal tumors where full surgical resection is challenging (4, 13, 14). Multimodal treatment with surgery, RT and chemotherapy may also be considered for canine HNC patients, particularly for cancers with significant risk of metastatic spread (13). Though veterinary RT facilities are small in size and number compared to human facilities, RT has increasingly become available for veterinary patients (15). Tumors of the head and neck in dogs and cats account for a large percentage of the neoplasms treated with RT in veterinary patients (15, 16). Recently, more precise RT techniques such as image guided RT and IMRT have also been used for many patients in veterinary medicine (14, 17).

Accurate definition of TVs and OARs is required for successful high precision RT (18), regardless of species. Tumor and/or organ volume contours can also be required for extraction of quantitative image-based features used in radiomics studies (19), where the primary aim is to identify imaging biomarkers. In clinical practice, TV and OAR definition is typically performed manually by clinical experts who contour the given structures on axial anatomical images, usually RT planning computed tomography (CT) images, using functional images as support if available. Manual contouring is, however, inherently subject to intra- and interobserver variability, introducing significant geometric uncertainties in RT planning and delivery (18). Inaccurate contour definitions can severely affect treatment outcome, potentially leading to underdosing of TVs and associated increased risk of locoregional failure or too high dose to normal tissues and subsequent increased RT toxicity (20–22). Furthermore, manual contouring is time and labor-intensive, particularly for HNC where the complexity and number of structures are considerable (23).

Recognizing the limitations of manual contouring, various automatic segmentation (auto-segmentation) methods and their potential application in the RT planning workflow have received significant attention. Over the past decade, deep learning methods have rapidly gained a central position within medical image analysis, particularly for semantic segmentation tasks such as contouring of RT structures. Many studies have shown that deep learning with convolutional neural networks (CNNs) can provide highly accurate auto-segmentations in human subjects, surpassing alternative segmentation methods (24–30). Moreover, the use of CNNs to guide manual contouring can decrease both contouring time and interobserver variability (31, 32). Several studies have evaluated the use of CNNs for segmentation of the gross tumor volume (GTV) or OARs in human HNC subjects, achieving high-quality segmentations based on RT planning CT, positron emission tomography (PET) and/or magnetic resonance (MR) images (29–31, 33–41). Even though there is increased focus on various deep learning applications in veterinary medicine, as exemplified by (42–46), few studies have evaluated the use of CNNs for semantic segmentation tasks in veterinary patients (47–49). Only two studies (48, 49) have focused on RT structures. Park et al. (48) used CNNs to contour various OARs in canine HNC patients (*n* = 90) based on CT images, obtaining similar segmentation performance as reported for humans, whereas Schmid et al. (49) applied CNNs to contour the medial retropharyngeal lymph nodes in CT images of canine HNC patients (*n* = 40) obtaining acceptable performance. Auto-segmentation of the GTV or any other TV has, to the best of our knowledge, not previously been explored for veterinary patients including dogs. Given the increased use of RT for canine HNC patients, it is highly warranted to investigate the applicability of automatic GTV segmentation in this group of patients.

One challenge for machine learning (in general) and deep learning (in particular) in the medical domain is that the number of available samples is often limited. Supervised CNN algorithms generally require large, labeled training sets. As the contouring process is laborious and must be done by a clinical expert to ensure satisfactory contour quality, it might not be feasible to label numerous images if this is not done prospectively at the time of treatment. Moreover, in the case of relatively rare diseases the number of available subjects will be low. Transfer learning has been proposed as a strategy to tackle limited training data (50).

The essence of transfer learning is to apply knowledge gained from solving one problem, referred to as the source problem, to solving a novel, separate problem, referred to as the target problem (50, 51). This approach has also been applied to deep learning-based medical segmentation tasks [for a summary, see (52)]. In veterinary science, transfer learning has been used successfully to segment acutely injured lungs in a limited number of CT images of dogs, pigs and sheep using a CNN model pretrained on a larger number of CT images of humans (47). These findings suggest that cross-species transfer learning from humans to dogs could potentially be used to increase the performance of other segmentation tasks such as GTV segmentation, particularly when the number of canine subjects is low (50).

The objective of the present study was to evaluate the applicability of CNNs for fully automatic segmentation of the GTV in canine HNC based on CT images. In addition, the impact of transfer learning from a larger cohort of human HNC patients on auto-segmentation performance was investigated. Two main approaches to model training were assessed: (i) training CNN models from scratch based solely on CT images of canine patients (*n* = 36), and (ii) using a transfer learning approach where CNN models were pretrained on CT images of human HNC patients (*n* = 197) and subsequently fine-tuned on CT images of canine patients. These two approaches were compared to a reference approach (iii) where CNN models trained solely on human data were applied directly to canine data, without transfer learning.

## 2. Materials and methods

In the present work, two different datasets consisting of contrast-enhanced CT images of canine and human patients, referred to as the canine and human datasets, respectively, were used to train CNN auto-segmentation models. Characteristics of the patients in the canine and human datasets can be found in Tables 1, 2, respectively. CT imaging and reconstruction parameters are summarized in Table 3.

**Table 1:** Patient characteristics of the canine dataset.

**Characteristics^a^**	**All patients (*n* = 36)**	**Fold 1 (*n* = 9)**	**Fold 2 (*n* = 9)**	**Fold 3 (*n* = 9)**	**Fold 4 (*n* = 9)**
**Age (years)**					
Mean (range)	7.7 (1.1–13.6)	8.3 (4.6–13.6)	7.9 (1.1–11.1)	8.2 (4.4–10.1)	6.2 (2.0–10.0)
**Sex**					
Female	13 (36 %)	2 (22 %)	4 (44 %)	4 (44 %)	3 (33 %)
Male	23 (64 %)	7 (78 %)	5 (56 %)	5 (55 %)	6 (67 %)
**Weight (kg)**					
Mean (range)	32.1 (8.2–74.5)	26.9 (13.0–38.9)	35.3 (8.8–74.5)	30.9 (8.2–45.4)	35.4 (14.8–52.0)
**Tumor site**					
Oral cavity	5 (14 %)	3 (33 %)	1 (11 %)	0 (0 %)	1 (11 %)
Nasal cavity	14 (39 %)	2 (22 %)	5 (56 %)	5 (56 %)	2 (22 %)
Nasopharynx	1 (3 %)	0 (0 %)	0 (0 %)	0 (0 %)	1 (11 %)
Other	16 (44 %)	4 (44 %)	3 (33 %)	4 (44 %)	5 (56 %)
**Nodal status**					
Node involvement	4 (11 %)	2 (22 %)	1 (11 %)	1 (11 %)	0 (0 %)
**GTV-T (cm** ^3^ **)**					
Mean (range)	69.7 (4.5–358.7)	50.0 (8.8–123.9)	57.0 (4.5–195.0)	48.3 (8.5–91.5)	123.4 (12.4–358.7)
**GTV-N (cm** ^3^ **)**					
Mean (range)	9.8 (0.002–38.1)	0.5 (0.03–1.1)	38.1 (NA)	0.002 (NA)	NA (NA)

a Percentages may not sum to exactly 100 due to rounding.

GTV-T, gross primary tumor volume; GTV-N, involved nodal volume (for patients with node involvement); NA, not applicable.

**Table 2:** Patient characteristics of the human dataset.

**Characteristic^a^**	**All patients (*n* = 197)**	**Training set (*n* = 126)**	**Validation set (*n* = 31)**	**Test set (*n* = 40)**
**Age (years)**				
Mean (range)	60.3 (39.9–79.1)	60.5 (39.9–78.9)	60.7 (48.4–79.1)	59.4 (43.0–77.0)
**Sex**				
Female	49 (25 %)	28 (22 %)	10 (32 %)	11 (28 %)
Male	148 (75 %)	98 (78 %)	21 (68 %)	29 (72 %)
**Tumor stage** ^b^				
T1/T2	96 (49 %)	61 (48 %)	15 (48 %)	20 (50 %)
T3/T4	101 (51 %)	65 (52 %)	16 (52 %)	20 (50 %)
**Nodal stage** ^b^				
N0	47 (24 %)	29 (23 %)	8 (26 %)	10 (25 %)
N1	23 (12 %)	15 (12 %)	4 (13 %)	4 (10 %)
N2	120 (61 %)	78 (62 %)	17 (55 %)	25 (62 %)
N3	7 (4 %)	4 (3 %)	2 (6 %)	1 (3 %)
**Tumor site**				
Oral cavity	17 (9 %)	10 (8 %)	4 (13 %)	3 (7 %)
Oropharynx	143 (73 %)	91 (72 %)	22 (71 %)	30 (75 %)
Hypopharynx	16 (8 %)	12 (10 %)	3 (10 %)	1 (3 %)
Larynx	21 (11 %)	13 (10 %)	2 (6 %)	6 (15 %)
**GTV-T (cm** ^3^ **)**				
Mean (range)	25.0 (0.8–285.0)	26.0 (0.8–285.0)	21.8 (0.8–78.2)	24.3 (1.4–157.6)
**GTV-N (cm** ^3^ **)**				
Mean (range)	24.3 (0.5–276.7)	27.5 (0.5–276.7)	17.7 (0.9–77.8)	19.5 (0.5–76.4)

a Percentages may not sum to exactly 100 due to rounding.

b Staging according to the 7th edition AJCC/UICC tumor-node-metastasis system.

GTV-T, gross primary tumor volume; GTV-N, involved nodal volume (for patients with nodal stage ≥ N1).

**Table 3:** CT imaging and reconstruction parameters.

**Human dataset (*n* = 197)**	
Scanner	Siemens Biograph 16, Siemens Healthineers GmbH, Erlangen, Germany
Scan mode	Helical (rotation time 0.5 s, pitch 0.75)
Peak tube voltage	120 kV
Reconstructed slice thickness	2.00 mm
Reconstruction kernel	B30f/B30s
Matrix size	512 × 512
Pixel size	0.98 × 0.98 mm^2^ (*n* = 161)
	1.37 × 1. 37 mm^2^ (*n* = 30)
	0.89 × 0.89 mm^2^ (*n* = 2)
	0.96 × 0.96 mm^2^ (*n* = 1)
	0.92 × 0.92 mm^2^ (*n* = 1)
	0.88 × 0.88 mm^2^ (*n* = 1)
	0.82 × 0.82 mm^2^ (*n* = 1)
Contrast agent	Visipaque 320 mg iodine/ml
**Canine dataset (*****n*** = **36)**	
Scanner	GE BrightSpeed S, GE Healthcare, Chicago, Illinois, USA
Scan mode	Helical (rotation time 1.0 s, pitch 0.75)
Peak tube voltage	120 kV
Reconstructed slice thickness	1.25 mm (*n* = 3)
	2.00 mm (*n* = 3)
	2.50 mm (*n* = 24)
	3.00 mm (*n* = 4)
	3.75 mm (*n* = 2)
Reconstruction kernel	Standard
Matrix size	512 × 512
Pixel size (range)	0.22 × 0.22 mm^2^ – 0.49 × 0.49 mm^2^
Contrast agent	Omnipaque 300 mg iodine/ml

### 2.1. Patients and imaging

#### 2.1.1. Canine dataset

The canine data was collected retrospectively by reviewing the imaging database and the patient record system of the University Animal Hospital at the Norwegian University of Life Sciences (NMBU). Potential patients were identified by searching the imaging database over the years 2004–2019, resulting in 1,304 small animal cases that were reviewed using the following inclusion criteria: canine patients with confirmed malignant neoplasia of the head or cervical region with a complete imaging examination including contrast-enhanced CT. A total of 36 canine cases met these criteria and were included in the canine HNC dataset. As these data were generated as part of routine patient workup, approval from the animal welfare committee was not required. Baseline CT imaging was performed pre and 1 min post intravenous contrast administration, using a GE BrightSpeed S CT scanner (GE Healthcare, Chicago, Illinois, USA). The animals were scanned in sternal recumbency under general anesthesia.

#### 2.1.2. Human dataset

The human data used in this study was obtained from a retrospective study of HNC patients with SCC of the oral cavity, oropharynx, hypopharynx, and larynx, treated with curative radio(chemo)therapy at Oslo University Hospital between 2007 and 2013 (53). The study was approved by the Regional Ethics Committee and the Institutional Review Board. ^18^F-fluorodeoxyglucose (FDG) PET/CT imaging was performed at baseline on a Siemens Biograph 16 (Siemens Healthineers GmbH, Erlangen, Germany) with a RT compatible flat table and RT fixation mask. Only the RT planning contrast-enhanced CT images were included in our present work and patients who did not receive contrast agent were excluded from the analysis, resulting in a dataset of 197 patients. This set of patients has previously been described and analyzed in two separate auto-segmentation studies (29, 34). Further details on the imaging protocol can be found in (29).

### 2.2. Manual GTV contouring

Manual GTV contours were used as the ground truth for training and evaluation of auto-segmentation models. For both datasets, manual contouring was performed in axial image slices and the GTV was defined to encompass the gross primary tumor volume (GTV-T) and any involved nodal volume (GTV-N) if present.

For the human patients, manual GTV contouring was done prospectively in the treatment planning system at the time of initial RT planning and in accordance with the previous DAHANCA Radiotherapy Guidelines (54). The manual contouring was based on both FDG PET and contrast-enhanced CT images. First, the GTV was contoured by an experienced nuclear medicine physician based on FDG PET findings. Next, one or two oncology residents refined the delineations based on contrast-enhanced CT images and clinical information. Finally, the delineations were quality assured by a senior oncologist.

Contouring of the canine GTVs was performed retrospectively by a board-certified veterinary radiologist (H.K.S.) with radiation oncology residency training. Contours were defined based solely on contrast-enhanced CT images using the 3D Slicer software (https://www.slicer.org) (55). The resulting delineations were smoothed in 3D Slicer using an in-plane median filter (5 × 5 kernel) before further image pre-processing. This was done to minimize the differences between the canine GTVs and the human GTVs, as the latter were smoothed by default in the hospital treatment planning system.

### 2.3. Image pre-processing

All CT images and corresponding manual GTV delineations were resampled to an isotropic voxel size of 1.0 × 1.0 × 1.0 mm^3^ to achieve a consistent voxel size and retain the actual anatomical size ratio between patients/species. Details regarding the resampling of the human HNC dataset can be found in (29). All other image pre-processing was performed using Python and SimpleITK (56).

The images of the human dataset were first cropped to a volume of interest (VOI) of size 191 × 265 × 173 mm^3^, defined to encompass the head and neck region. Subsequently, the canine images were cropped and/or padded symmetrically about each axis to obtain the same image dimensions as the above VOI while keeping the patient in the center of each 3D image stack. If padding was applied, added voxels were given a value corresponding to background/air.

### 2.4. Deep learning architecture and model training

**Figure 1:**
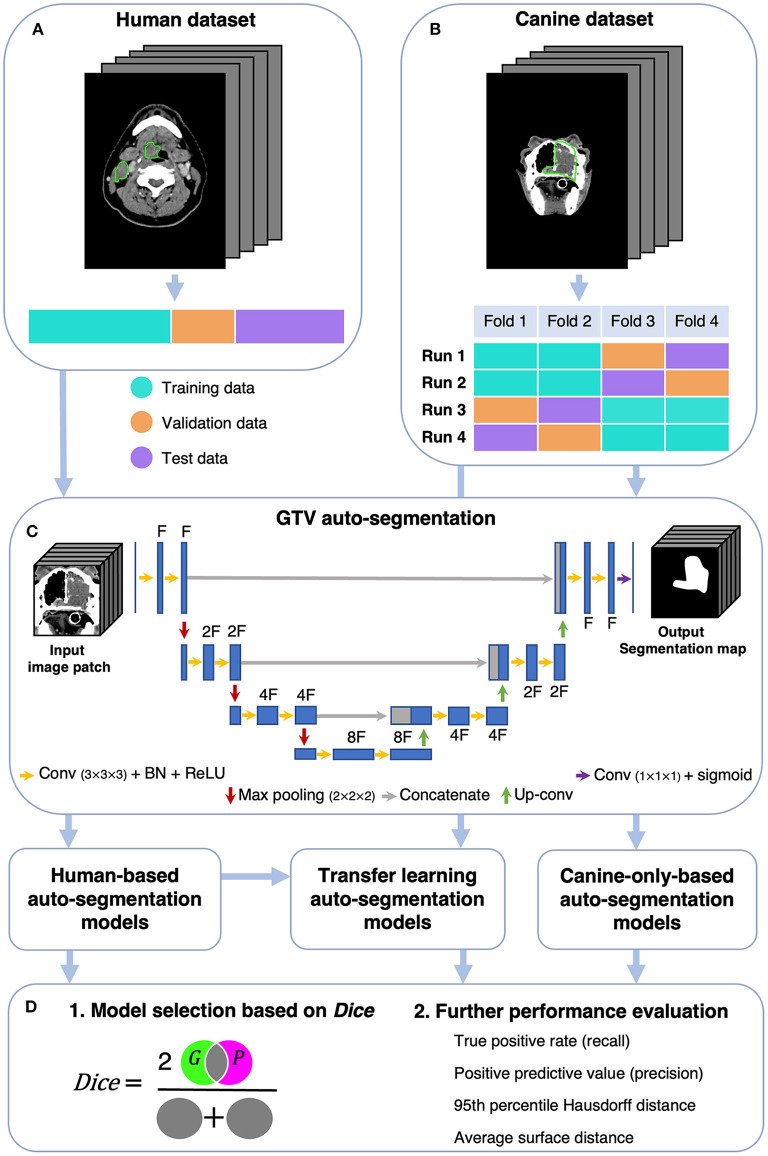
Schematic overview of the analysis. The human **(A)** and canine **(B)** datasets consisted of CT images and corresponding manual GTV delineations (green contours) cropped to a volume of interest of 191 × 265 × 173 mm^3^. The human dataset was divided into a training, validation, and test set, whereas the canine dataset was divided into four folds used for model training and evaluation. **(C)** GTV auto-segmentations were generated using a 3D U-Net architecture with input image patches of size 112 × 112 × 112 mm^3^ [shown U-Net: depth of 3 (3 max pooling operations) and F filters in the first convolutional layer]. Auto-segmentation models were trained on either human or canine data, where the model trained on human data was further used for transfer learning (fine-tuning with canine data). **(D)** Model performances were first assessed using the Dice similarity coefficient [*Dice*; cf. Equation (1), Section 2.5], measuring the overlap between manual ground truth delineations (*G*) and predicted auto-segmentations (*P*). The models with superior *Dice* characteristics were selected for further performance evaluation. CT, computed tomography; GTV, gross tumor volume; Conv, Convolution; BN, Batch Normalization; ReLU, Rectified Linear Unit; Up-conv, Up-convolution.

Canine auto-segmentations were obtained using two main approaches, namely (i) by training CNN models from scratch based on the canine dataset only, and (ii) a transfer learning approach where CNN models were pretrained on the human dataset and subsequently fine-tuned on the canine dataset. As a comparison to the above approaches, the CNN models trained on the human dataset only were evaluated directly on the canine dataset (i.e., without transfer learning). A schematic overview of the analysis is given in Figure 1. Note that all CNN models were based only on contrast-enhanced CT images, as no PET images had been acquired for the canine patients.

A 3D U-Net CNN architecture (57) with the Dice loss function (58) was used throughout this study. All models were trained using the Adam optimizer with an initial learning rate of 10^−4^ (59). Further details about the CNN architecture are outlined in Figure 1C. Experiments were run on the Orion High Performance Computing resource at NMBU using deoxys, our in-house developed Python framework for running deep learning experiments with emphasis on TV auto-segmentation (https://deoxys.readthedocs.io/en/latest/).

We assessed the impact of varying the following: (1) the complexity of the U-Net architecture, (2) the CT window settings of the input images, and (3) the training set image augmentation configurations. First, for the models trained from scratch on canine data, different U-Net complexities were assessed using network depths of 3, 4 and 5 with a corresponding number of filters in the first network layer of 32, 64 and 64. Second, we explored using CT window settings with a window center equal to the median Hounsfield unit (HU) value within the ground truth GTV voxels of the relevant training data (human training set: 65 HU; canine training sets: 93 HU (folds 1 and 2) and 96 HU (folds 3 and 4)) and a window width of either 200 HU or 400 HU. CNN models were trained using either one single input channel with windowed CT images, two separate input channels consisting of CT images with and without windowing, or three input channels where two were with different window settings according to the canine and human training data, and the third channel consisted of CT images without windowing. Third, the following image augmentation configurations were evaluated: no image augmentation, image augmentation in the form of 3D rotation, zooming, and flipping, or 3D elastic deformations. Code for running the experiments, including the above image augmentation schemes, is available at https://github.com/argrondahl/canine.

To train models and evaluate model performance, the datasets were divided as follows: patients in the human dataset were split into a training (*n* = 126), validation (*n* = 31) and test (*n* = 40) set (Figure 1A) using randomly stratified sampling to obtain similar primary tumor stage distributions in each set (cf. Table 2; staging according to the 7th edition AJCC/UICC tumor-node-metastasis system). Patients in the canine dataset were randomly divided into four equally sized folds (*n* = 9). Following the cross-validation and test set evaluation strategy outlined in Figure 1B, each of these folds was used twice for model training (cyan), once as a validation set (orange) and once as a test set (purple). With this strategy, each canine model configuration was trained four times, and each patient was twice in the training set, once in the validation set and once in the test set. Thus, the validation and test set performances could be calculated for each of the 36 patients. This procedure was chosen to acquire a robust estimate of the auto-segmentation performance despite a limited number of canine patients, taking individual differences across patients into account and making the validation and test set performances less dependent on how the data was split.

Most models were trained for 100 epochs, saving model weights (checkpointing) to disc every epoch. However, for the pretraining of models on human data, early stopping with patience 30 (i.e., stop training if validation loss does not improve for 30 consecutive epochs) was used to avoid overfitting to the source domain. For continued training (fine-tuning) of pretrained models, we compared initializing the Adam optimizer with an initial epoch set to 50 vs. 100. After training of one model, the optimal epoch was identified as the epoch maximizing the mean per patient Sørensen-Dice similarity coefficient (60, 61) (*Dice*; cf. Section 2.5 below) on validation data.

### 2.5. Performance evaluation

The quality of the CNN-generated auto-segmentations were first assessed using *Dice* (60, 61), which is a volumetric overlap metric quantifying the degree of spatial overlap between the set of voxels in the ground truth *G* and the predicted auto-segmentation *P* (Figure 1D). *Dice* is defined as:


(1) $$\begin{array}{l} Dice\;=\;\frac{2\;|P\cap G|}{\left|P|+|G\right|}=\frac{2TP}{2TP+FP+FN}, \end{array}$$

where *TP, FP* and *FN* refer to the true positive, false positive, and false negative voxels, respectively. *Dice* ranges from 0 to 1, where 0 corresponds to no overlap and 1 corresponds to perfect overlap between the sets. Based on the *Dice* performances on validation and test data, we selected one model trained from scratch on canine data and one model trained with transfer learning for more in-depth performance evaluation and comparison.

As *Dice* does not separate between *FP* and *FN* voxels and is known to be volume-dependent, the auto-segmentation performance of the two selected models were further assessed using the positive predictive value (*PPV*), the true positive rate (*TPR*), the 95th percentile Hausdorff distance (*HD*_95_) (62) and the average surface distance (*ASD*) (63).

*PPV* and *TPR*, commonly also referred to as precision and recall, are defined as:


(2) $$\begin{array}{l} PPV\;=\frac{TP}{TP+FP}, \end{array}$$

and


(3) $$\begin{array}{l} TPR\;=\;\frac{TP}{TP+FN}. \end{array}$$

As seen from Equations (2) and (3), *PPV* is the fraction of the predicted auto-segmentation *P* that overlaps with *G*, while *TPR* is the fraction of the ground truth *G* that overlaps with *P*. In the context of TVs used for RT, *PPV* measures the degree of avoiding inclusion of normal tissue voxels in the auto-segmentation, while *TPR* measures the degree of target coverage.

The distance metrics were calculated from the two sets of directed Euclidian distances between the surface voxels of *P* and *G* (set 1: all distances from *P* to *G*; set 2: all distances from *G* to *P*). The *HD*_95_ and *ASD* were then defined as the maximum value of the 95th percentiles and averages, respectively, of the above two sets of surface distances. *HD*_95_ reflects the largest mismatch between the surfaces of *P* and *G*, whereas *ASD* is used to quantify the typical displacement between the two surfaces. These metrics should both be as small as possible.

The above performance metrics were calculated per patient, based on all voxels in the pre-defined 3D VOI (cf. Section 2.3). The Python deepmind library was used for calculation of surface-distance-based metrics (https://github.com/deepmind/surface-distance).

## 3. Results

**Figure 2:**
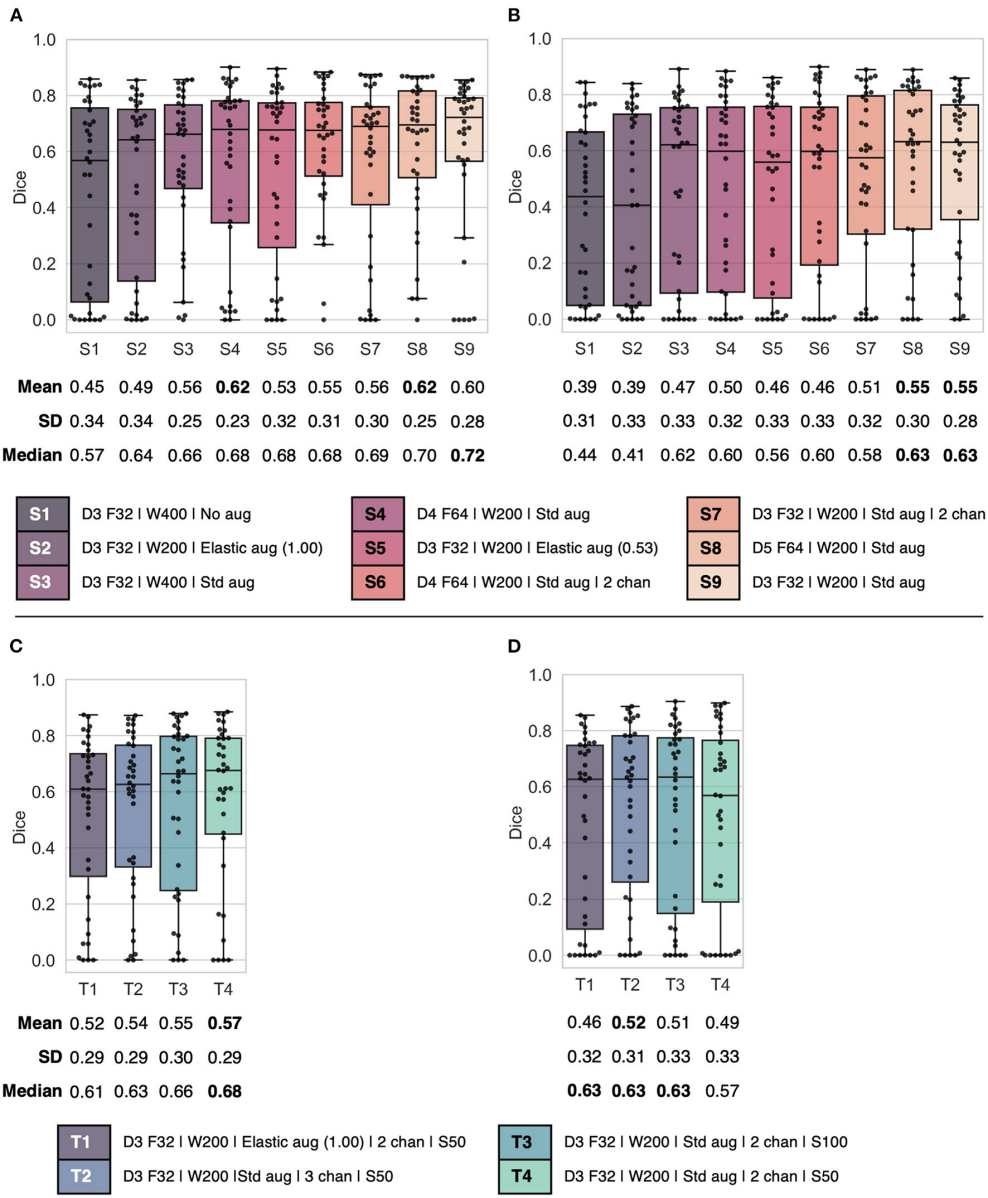
Combined box and swarm plots of per patient *Dice* scores for different model configurations, showing each patient as a separate data point (black). Top: **(A)** validation and **(B)** test results for models S1–S9 trained from scratch on canine data. Bottom: **(C)** validation and **(D)** test result for models T1–T4 trained using the transfer learning approach. Model configurations (S1–S9 and T1–T4) are as follows: Model complexity given by U-Net depth D and number of filters F in the first layer; CT window setting with window width W in HU; Image augmentation settings (Std aug: zooming, rotation and flipping; Elastic aug: elastic deformation on a proportion (0.53 or 1.00) of the training set images); Number of input channels (chan), default 1 channel unless otherwise stated; Initial epoch setting S, either 50 or 100 epochs (transfer learning only). SD, standard deviation.

The validation and test set *Dice* performances of canine models trained with varying network complexity, CT window settings, number of input channels, and image augmentation schemes are summarized in Figure 2. Models trained from scratch (Figures 2A, B) resulted in mean validation and test set *Dice* scores in the range 0.45–0.62 and 0.39–0.55, whereas models trained with transfer learning (Figures 2C, D) resulted in validation and test set *Dice* scores ranging from 0.52 to 0.57 and 0.46 to 0.52. In comparison, when evaluated on human data the pretrained human-based models resulted in mean validation and test set *Dice* scores of 0.46–0.55 and 0.48–0.54. Models trained on human data only and evaluated directly on canine data resulted in unacceptably low mean *Dice* test scores of 0.02–0.08, even though some models achieved relatively high *Dice* scores for some patients (range of maximum *Dice* per model: 0.16–0.67) (data not shown).

**Figure 3:**
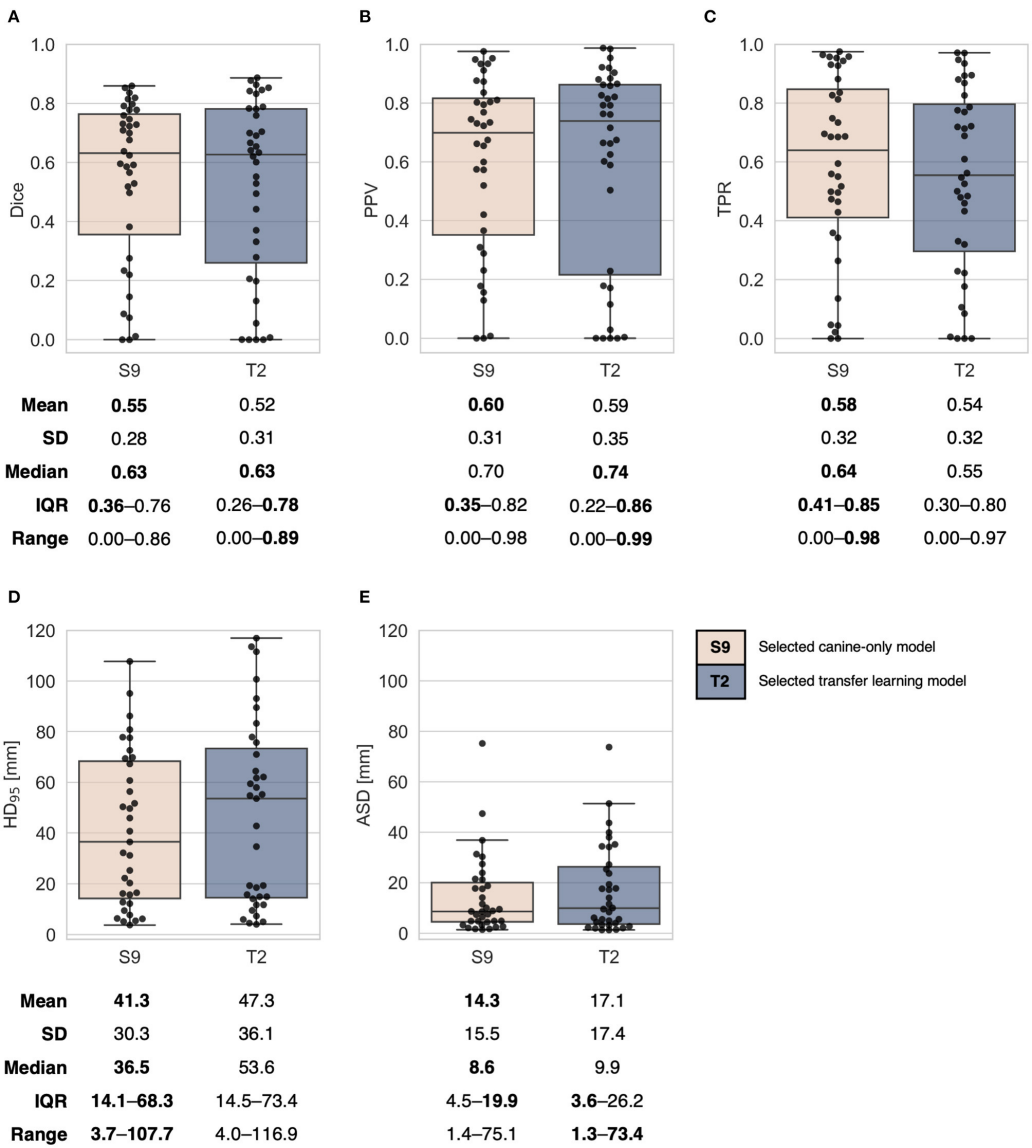
Combined box and swarm plots of per patient auto-segmentation performance metrics for the two selected models trained from scratch on canine data (S9, Figures 2A, B) and trained using the transfer learning approach (T2, Figures 2C, D). Each patient is shown as a separate data point (black). Performance metrics: **(A)** Dice similarity coefficient (*Dice*), **(B)** positive predictive value (*PPV*), **(C)** true positive rate (*TPR*), **(D)** 95th percentile Hausdorff distance (*HD*_95_), **(E)** average surface distance (*ASD*). The exact positioning of individual data points in **(A)** may differ from the respective plots in Figures 2B, D, due to randomness in the swarm plots. SD, standard deviation; IQR, interquartile range. One patient without any predicted auto-segmentation was excluded from calculations of *HD*_95_ **(D)** and *ASD* **(E)**.

For models trained from scratch on canine data, the highest mean validation *Dice* score (0.62) was observed for models S4 and S8 (Figure 2A), which both used one input channel with a narrow CT window width (200 HU), standard image augmentation (flipping, rotation, zooming) and a high model complexity (depth of 4 and 5, respectively, and 64 filters in the first layer). On the other hand, the less complex model S9 (depth of 3 and 32 filters in the first layer), which was otherwise identical to models S4 and S8, showed comparable mean validation *Dice* performance (0.60) and the highest median *Dice* (0.72). Moreover, models S8 and S9 resulted in similar overall test set *Dice* performances, whereas model S4 had poorer performance on test data (Figure 2B). As model S9 was the least complex and, therefore, the least resource-demanding to train, while at the same time providing competitive *Dice* performance, it was selected for further performance evaluation (Figure 3) and the given complexity and CT window width was used for the transfer learning experiments.

For the transfer learning models, the highest mean validation *Dice* score (0.57) was observed for model T4 (Figure 2C; depth of 3 with 32 filters in the first layer, CT window width of 200 HU, 2 input channels with window center derived from (1) human and (2) canine training data, standard image augmentation and initial epoch set to 50). However, model T2, which included an additional CT channel with no windowing, but was otherwise the same as model T4, displayed the highest test set mean *Dice* (0.52) and a favorable test set *Dice* interquartile range (Figure 2D), indicating a moderately better ability to generalize to previously unseen data. Thus, among the transfer learning models, model T2 was selected for computation of additional performance metrics (Figure 3).

The two selected models (S9 and T2, Figure 2) generally showed similar auto-segmentation performances on test data, as indicated by the plots and summary statistics of Figure 3. For both models, there was substantial inter-patient variation in the resulting auto-segmentation quality. The model trained from scratch on canine data resulted in the best mean performances for all included metrics. In general, the canine-only model had larger tumor coverage (higher mean and median *TPR*) but tended to include more normal tissue (lower median *PPV*) than the transfer learning model. The transfer learning model did, however, achieve the highest per patient overlap with ground truth contours (maximum *Dice*: 0.89) and the lowest per patient *ASD* (minimum *ASD*: 1.3 mm). In addition, the transfer learning model resulted in a higher number of very high-quality auto-segmentations (*Dice* ≥ 0.85; *n* = 5) than the model trained from scratch (*n* = 2). However, the transfer learning model tended to perform the poorest on more difficult-to-segment canine patients, as reflected by the poorer first quartile *Dice, TPR, PPV* and *HD*_95_ values.

**Figure 4:**
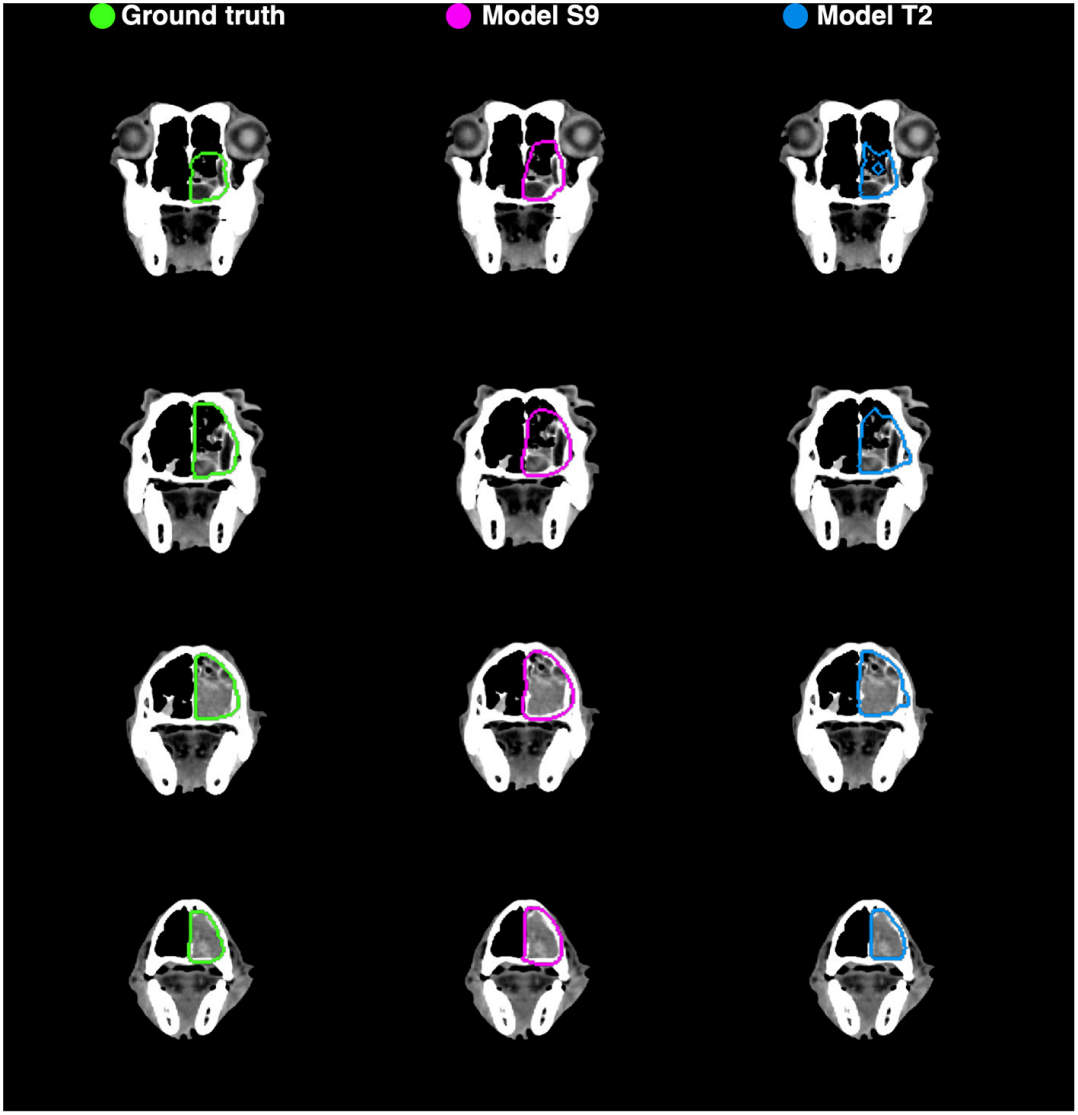
Manual ground truth and automatic deep learning generated gross tumor volume contours in four CT image slices from one canine test set patient (nasal cavity tumor). **(Left column)** Manual ground truth contours (green). **(Middle column)** Auto-segmentation generated by model S9 (magenta; model trained from scratch on canine data only). **(Right column)** Auto-segmentation generated by model T2 (blue; model trained using transfer learning). The two models resulted in Sørensen-Dice similarity coefficients of 0.85 (model S9) and 0.89 (model T2) for the given patient (calculated over all 173 image slices).

**Figure 5:**
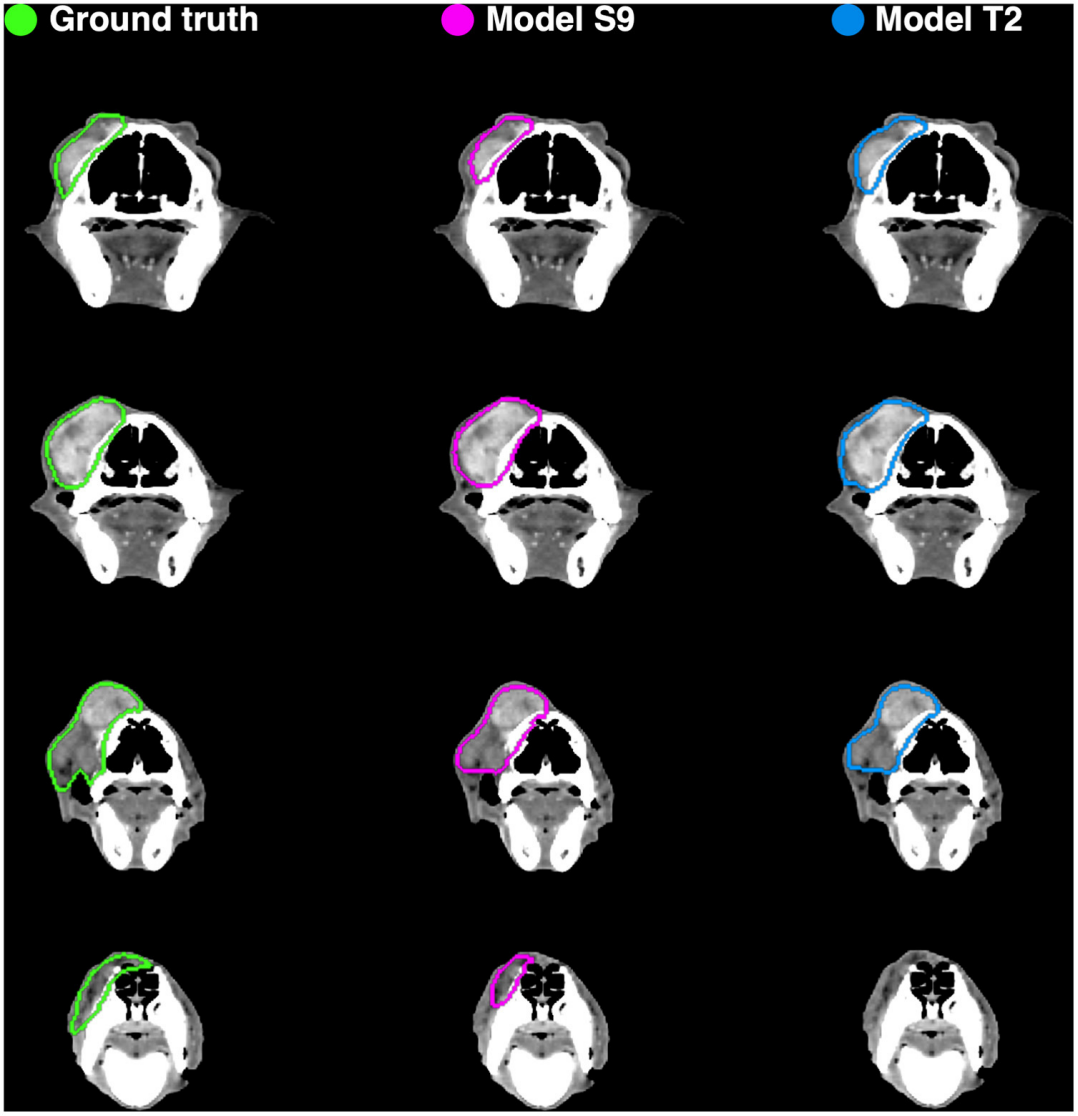
Manual ground truth and automatic deep learning generated gross tumor volume contours in four CT image slices from one canine test set patient (sarcoma). **(Left column)** Manual ground truth contours (green). **(Middle column)** Auto-segmentation generated by model S9 (magenta; model trained from scratch on canine data only). **(Right column)** Auto-segmentation generated by model T2 (blue; model trained using transfer learning). The two models resulted in Sørensen-Dice similarity coefficients of 0.86 (model S9) and 0.84 (model T2) for the given patient (calculated over all 173 image slices).

**Figure 6:**
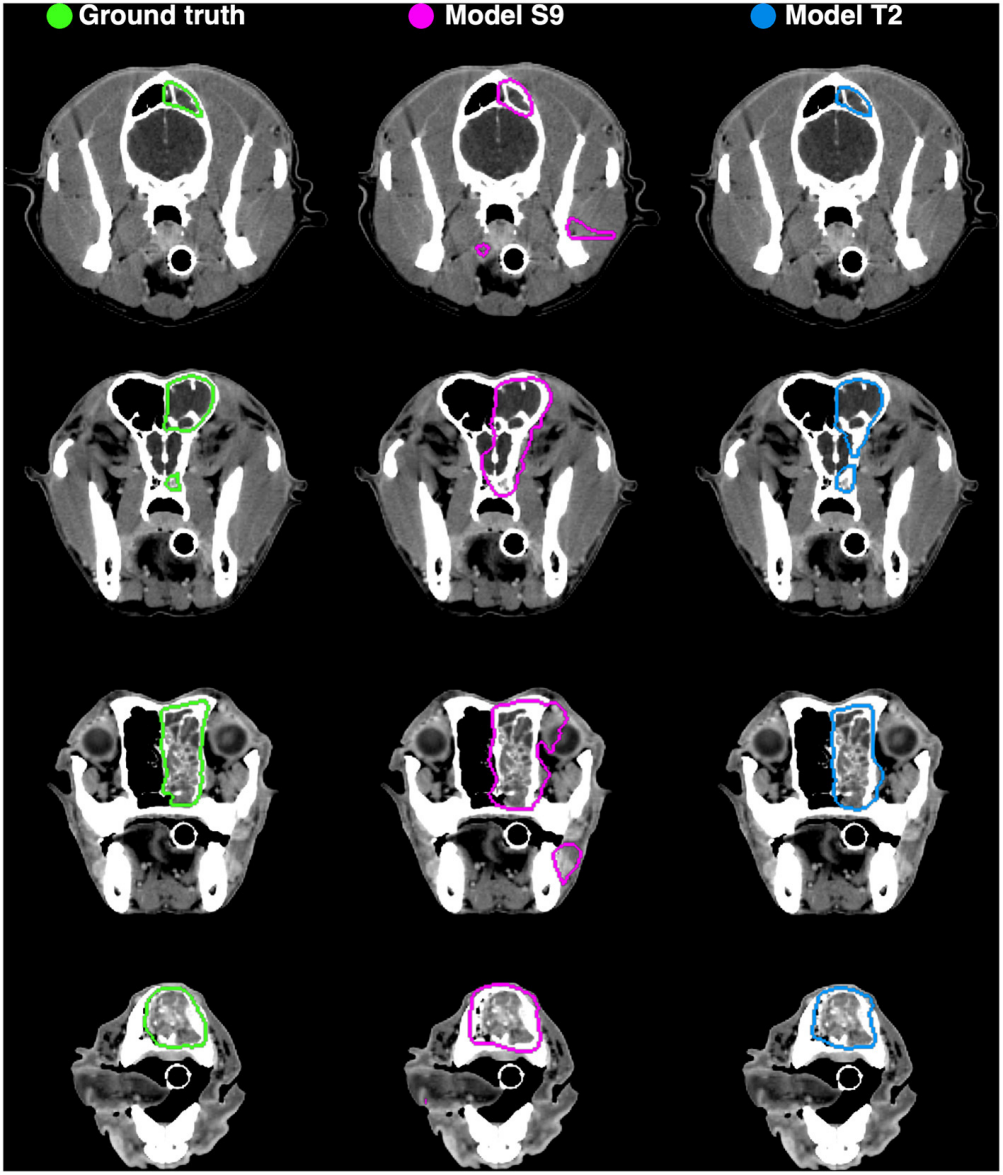
Manual ground truth and automatic deep learning generated gross tumor volume contours in four CT image slices from one canine test set patient (nasal cavity tumor). **(Left column)** Manual ground truth contours (green). **(Middle column)** Auto-segmentation generated by model S9 (magenta; model trained from scratch on canine data only). **(Right column)** Auto-segmentation generated by model T2 (blue; model trained using transfer learning). The two models resulted in Sørensen-Dice similarity coefficients of 0.72 (model S9) and 0.85 (model T2) for the given patient (calculated over all 173 image slices).

Example auto-segmentations are shown in Figures 4–6. In general, the two selected models achieved the highest quality auto-segmentations for patients with nasal cavity tumors, which was the most frequently occurring tumor site in the canine dataset. The canine-only and transfer learning models both achieved a mean test set *Dice* of 0.69 for nasal cavity tumors, compared to the corresponding *Dice* scores of 0.55 and 0.52 for all tumor sites. As exemplified in Figure 4, tumor regions with relatively homogeneous HU values within the ground truth were generally easier to segment correctly. High quality auto-segmentations were also seen for other tumor sites where the tumor was distinct from the surrounding normal tissues and clearly affected the anatomical shape/boundary of the animal (Figure 5). Peripheral parts of the GTV were often more difficult to segment than central parts (Figure 5; bottom row). In some cases, the auto-segmentations included substantial normal tissue regions due to over-estimation of the GTV boundaries or prediction of separate smaller false positive structures. False positive structures and inclusion of particularly brain and eye tissues in the predicted auto-segmentation were more pronounced for the model trained only on canine data (S9). An example is shown in Figure 6.

Both models resulted in poor auto-segmentations for patients with atypical tumor sites, atypical GTV shapes and/or a substantial number of image slices with atypical/very heterogeneous HU values inside the ground truth GTV. Neither of the models was able to successfully segment the smaller canine GTV-N structures. The above patterns indicate that the auto-segmentation performance was dependent on the number of representative canine training samples, regardless of model training approach.

## 4. Discussion

This is the first study to evaluate deep learning-based auto-segmentation of TVs for RT in veterinary patients. Although dogs display breed-related variation in the head and neck anatomy and size, which could potentially complicate the auto-segmentation task, our results show that CNNs can provide high-quality GTV auto-segmentations for this group of patients, despite a limited number of training samples. Our two main approaches, namely (i) CNN models trained from scratch on canine data or (ii) CNN models pretrained on human HNC patients and fine-tuned using canine patients (transfer learning), generally gave similar results. In both cases the mean overlap with the expert ground truth contours was similar to what is obtained for human HNC patients.

Previous studies on human HNC subjects report mean validation and/or test set *Dice* scores in the range of 0.31–0.66 for CNN-generated auto-segmentations of the GTV based on CT images (29, 33, 34, 64). The relatively large variation in reported performances is likely related to differences in image pre-processing, such as CT window settings and VOI dimensions, the composition of the datasets and/or CNN architecture. Of the above studies, the highest mean *Dice* [0.66; cross-validation result (29)] was obtained using a 2-dimensional (2D) U-Net architecture and a considerably smaller pre-defined VOI than in our present work. Moe et al. (34) obtained a mean test set *Dice* of 0.56 using the same 2D U-Net architecture on larger image VOIs encompassing the entire head and neck region but excluding image slices without any ground truth delineation. Both Groendahl et al. (29) and Moe et al. (34) used the same single-center HNC patients as in our present study. The lowest mean *Dice* scores [0.31 (33) and 0.49 (64)] were reported for auto-segmentation in multi-center patient cohorts, which is generally more challenging than single-center segmentation, using wider CT window widths. Both latter studies used similarly sized image VOIs and 3D architectures, which are generally superior to their 2D counterparts, as in or present work. In comparison to the above human studies, our best-performing canine models trained from scratch or with transfer learning, both using a 3D U-Net architecture and a narrow CT window, resulted in similar or higher mean validation (test) *Dice* scores of 0.62 (0.55) and 0.57 (0.52), respectively, compared to the above studies. The *Dice* performances of our CNN models were also comparable to the reported *Dice* agreement (0.56–0.57) between clinical experts performing manual GTV contouring in human HNC patients based solely on CT images (65, 66).

Human cancer patients normally undergo several imaging procedures as part of diagnosis and treatment planning. It is also common to base contouring of the GTV on multimodal image information. Thus, most of the recent studies on GTV segmentation in human HNC patients investigate using multimodality images as input to the network for increased performance. As PET/CT imaging is becoming more common in veterinary medicine (67), it is worth noting that all the above human HNC studies reported significant increases (range: 12–129 %; median: 25 %) in mean *Dice* scores when using both FDG PET and CT images as CNN model input. Similar improvements are likely possible for canine patients, provided that the lesions are comparably FDG PET avid. PET imaging is, however, not likely to become widely available for veterinary patients in the near future. A more realistic approach at present would be to investigate the potential added benefit of including both pre and post contrast CT images as input to CNN models trained from scratch on canine data. In the present work, however, we chose to focus solely on post contrast CT images as these images were also available for the human patients.

As HNC is a heterogeneous group of cancers, many studies on human HNC subjects focus only on one anatomical primary tumor site. Specifically oropharyngeal cancer which is one of the most frequently occurring HNC sites in humans worldwide (2), or nasopharyngeal cancer which display very distinctive properties, are commonly analyzed separately (31, 36, 41, 64, 68–72). A similar approach could be beneficial for further analyses of auto-segmentation of the GTV in canine HNC subjects. In our present work, the highest quality auto-segmentations were generally obtained in patients with nasal cavity tumors. This is likely influenced by the tumor site distribution in our dataset, where this was the most frequent site. However, nasal cavity tumors display distinctive characteristics in terms of shape and location and generally have high contrast between tumor tissue and normal tissues/background, all of which could aid auto-segmentation. GTV segmentation is also particularly relevant for this group of canine HNC patients, as RT is indicated as the primary treatment (4, 13, 14).

Even though our results show that deep learning can provide high-quality GTV auto-segmentations in canine HNC patients, there are currently several limitations to this approach that must be resolved to increase its potential clinical usefulness. First, regardless of model training approach, the auto-segmentation quality was variable between patients. The poorest performance was seen for patients with rare tumor sites and GTVs with atypical shapes or heterogeneous HU intensity values. This could be alleviated by having a larger training set where all tumor sites are represented to a greater extent. Another possibility, as outlined in the previous paragraph, is to focus on each tumor site separately. Furthermore, inclusion of both pre and post contrast CT images as model input may mitigate the issue of heterogeneous tumor intensities in some cases, as it can be relevant whether the heterogeneity is due to inherent anatomical factors or heterogeneous contrast enhancement. However, GTVs with very heterogeneous HU intensity values including relatively large proportions of bone and/or air might still be difficult to automatically segment correctly and would likely require intervention by a human expert. Secondly, the auto-segmentations could encompass false positive regions including OARs such as the eye and brain. To limit the need for human revision, smaller false positive structures could be removed in a post-processing step, using for example morphologic operations, whereas inclusion of OAR regions due to over-estimation of GTV boundaries could be reduced by combining OAR and TV segmentation. Segmentation of normal tissue structures such as OARs typically achieve higher *Dice* scores than TV segmentation, as organ shapes, locations and intensities generally are less variable between patients than tumors, though some OARs are more difficult to segment than others due to, e.g., poor CT contrast. Reported mean *Dice* scores of CT-based OAR segmentation using deep learning are 0.78–0.87 (30, 38–40) and 0.83 (48) for human and canine HNC patients, respectively, when averaged over various organ structures. Deep learning-based OAR segmentation may be considered clinically applicable for several OARs (37, 73) and is currently commercially available for RT in humans.

Transfer learning provided high performance but did not improve the mean performance metrics compared to training canine models from scratch. There are several potential factors that can contribute to why transfer learning did not outperform training from scratch, specifically related to the differences between the human and canine datasets. First, there are obvious anatomical differences between the human and canine head and neck region that might not be overcome by the use of image augmentation and fine-tuning of the pretrained human model. Second, the presence and degree of nodal involvement was significantly higher for the human patients. The majority of the human patients (76%) had known nodal involvement and the mean GTV-N size was similar to the mean GTV-T size, whereas few canine patients (11%) had known nodal involvement and the GTV-N structures were all small in size compared to the GTV-T. Third, the anatomical tumor site and cancer subtype distributions were not comparable between the two species. Fourth, the ground truth GTV contours were delineated under different conditions. Fifth, the CT imaging was conducted using different scanners with different imaging and reconstruction parameters. Regardless of the above differences between source and target domains, the transfer learning approach resulted in the highest per patient *Dice* score and to a greater extent avoidance of OARs. Thus, there is reason to assume that some features learned in the source domain were useful in the target domain, but that the usefulness was variable among the canine subjects.

A recent thorough investigation of transfer learning for different deep learning-based medical image segmentation tasks in humans, conducted by Karimi et al. (50), shows that transfer learning in general primarily decreased the training time for the target task and that improvements in auto-segmentation performance often was marginal and largely relied on the data and task. According to their results, statistically significant effects of transfer learning only occurred when the number of target training samples was low (~3–15 subjects). In other cases, models trained from scratch and transfer learning models were comparable in terms of auto-segmentation quality. Cross-species transfer learning was not evaluated in Karimi et al. (50) but our results are in line with their findings for transfer learning between human domains and tasks. Gerard et al. (47) applied transfer learning to segment acutely injured lungs in CT images of dogs, pigs, and sheep, obtaining median Jaccard index scores ≥0.90, which corresponds to *Dice* scores ≥0.95, using a multi-resolution CNN model pretrained on CT images of humans without acutely injured lungs. Their proposed transfer learning method was, however, not compared to training models from scratch on the target domain. Thus, the effect of transfer learning was not quantified, and the high performance could be related to the task or influenced by the CNN configuration rather than the transfer learning approach.

To summarize, segmentation of the GTV in canine and human HNC patients is an inherently challenging task. In this study, CNN models for auto-segmentation of the GTV in canine HNC patients, trained either from scratch on canine data or by using a cross-species transfer learning approach, provided promising results with high performance metrics comparable to results achieved in human HNC auto-segmentation studies. Our results show that transfer learning has the potential to increase segmentation performance for some patients, but differences between source and target domains as well as the heterogeneity of the disease within species can complicate the modeling. Therefore, care must be taken when transferring auto-segmentation models between species.

## Data availability statement

The datasets presented in this article are not readily available because access to the human dataset requires approval by the Regional Ethics Committee. The canine raw data supporting the conclusions of this article will be made available by the authors, without undue reservation. Requests to access the datasets should be directed to CMF, cecilia.futsaether@nmbu.no.

## Ethics statement

The studies involving human participants were reviewed and approved by the Regional Ethics Committee (REK) and the Institutional Review Board. Exemption from study-specific informed consent was granted by REK as this is a retrospective study and the patients are de-identified. Written informed consent for participation was not required for this study in accordance with the national legislation and the institutional requirements. Ethical review and approval was not required for the animal study because the image data were generated as part of routine patient workup. Written informed consent for participation was not obtained from the owners because the data was collected retrospectively and the patients were de-identified prior to analysis.

## Author contributions

ARG: conceptualization, methodology, software, data curation, formal analysis, visualization, writing—original draft preparation, and writing—review and editing. BNH: conceptualization, methodology, software, and writing—review and editing. OT and ÅS: conceptualization, methodology, and writing—review and editing. ED: data curation, funding acquisition, and writing—review and editing. EM and HKS: conceptualization, methodology, data curation, funding acquisition, and writing—review and editing. CMF: conceptualization, methodology, funding acquisition, project administration, supervision, and writing—review and editing. All authors contributed to the article and approved the submitted version.
